# Current status of research on exosomes in general, and for the diagnosis and treatment of kidney cancer in particular

**DOI:** 10.1186/s13046-021-02114-2

**Published:** 2021-09-28

**Authors:** Weipu Mao, Keyi Wang, Zonglin Wu, Bin Xu, Ming Chen

**Affiliations:** 1 Department of Urology, Shidong Hospital of Yangpu District, No. 999 Shiguang Road, Yangpu District, Shanghai, 200438 China; 2 Department of Urology, Affiliated Zhongda Hospital of Southeast University, No. 87 Dingjiaqiao, Hunan Road, Gulou District, Nanjing, 210009 China

**Keywords:** Biomarker, Exosomes, Renal cell carcinoma, Therapy

## Abstract

Kidney cancer is a common urological tumour. Owing to its high prevalence and mortality rate, it is the third most malignant tumour of the urinary system, followed by prostate and bladder cancers. It exerts a high degree of malignancy, and most of the distant metastasis occurs at an early stage; it is insensitive to chemoradiotherapy and easily develops drug resistance. The current treatment for kidney cancer mainly includes surgery, interventional embolization and targeted therapy; however, the treatment efficacy is poor. In recent years, the role of exosomes as mediators of intercellular communication and information exchange in the tumour microenvironment in tumour pathogenesis has attracted much attention. Exosomes are rich in bioactive substances such as nucleic acids, proteins and lipids and are involved in angiogenesis, immune regulation, drug resistance, formation of pre-metastatic niche, invasion and metastasis. This article reviews the ongoing research and applications of exosomes for the diagnosis and treatment of kidney cancer.

## Background

Kidney cancer, also known as renal cell carcinoma (RCC), is one of the most common malignancies of the urinary tract, and its incidence has increased at a rapid rate of 2% per year over the past two decades [1, 2]. In 2018, approximately 400,000 new cases and 170,000 deaths owing to kidney cancer were reported worldwide [3]. In 2015, approximately 74,000 new cases and 27,000 deaths owing to kidney cancer were reported in China [4]. Kidney cancer is insensitive to radiotherapy and chemotherapy, and surgery remains the mainstay of treatment for kidney cancer. However, approximately 30% of patients with kidney cancer develop metastasis on initial diagnosis, and approximately 25% of patients with localized kidney cancer may develop local recurrence or distant metastasis after surgery [5, 6]. Owing to recurrence or distant metastasis, the 5-year survival rate of patients with advanced kidney cancer is extremely low, approximately 5–10% [7, 8].

Exosomes are small extracellular vesicles composed of a lipid bilayer membrane structure; they are actively secreted by normal and cancer cells in the body and contain proteins, nucleic acids, lipids and other bioactive substances [9, 10]. Exosomes play an important role in the exchange of information between cells by releasing bioactive substances that fuse with receptor cell membranes or bind to cell surface receptors [11, 12]. Studies have demonstrated that exosomes play an important role in the development, diagnosis and treatment of kidney, prostate, bladder and breast cancers and serve potential clinical applications as tumour markers, therapeutic targets and drug nanocarriers in clinical settings [13–15].

This article reviews the ongoing research and applications of exosomes for the diagnosis and treatment of kidney cancer.

## Overview of exosomes

Exosomes are nanoscale biological vesicles released into surrounding body fluids upon fusion of multivesicular bodies and the plasma membrane; they are produced and secreted autonomously by living cells in vivo and are the smallest extracellular vesicles [16, 17]. Exosomes are subgroups of extracellular vesicles with an average diameter of about 30–100 nm [18, 19]. Exosomes originate from the intracellular body structure, which influences the composition of exosome contents after interaction with other intracellular vesicles and organelles [20]. Exosomes were previously considered non-functional substances until 2007, when it was discovered that exosomes may act as ‘messengers’ that carry genetic material for the exchange of intercellular information and act within the recipient cells, suggesting that exosomes can be involved in intercellular information exchange [21–23]. The membrane structure of exosomes is resistant to exogenous proteases and RNA enzymes, thus resulting in more stable intracellular functional proteins, messenger RNAs (mRNAs) and microRNAs (miRNAs) that make exosomes a sensitive marker for disease diagnosis [24, 25]. In many diseases, exosomes can function by altering cellular or tissue states, and exosome-related assays can be used as effective and non-invasive methods for disease diagnosis and monitoring [9, 10]. In addition, the study of molecular mechanisms related to exosome-mediated intercellular material exchange will also provide a theoretical basis for the development of exosome-related therapies [26, 27].

### Composition of exosomes

**Fig. 1:**
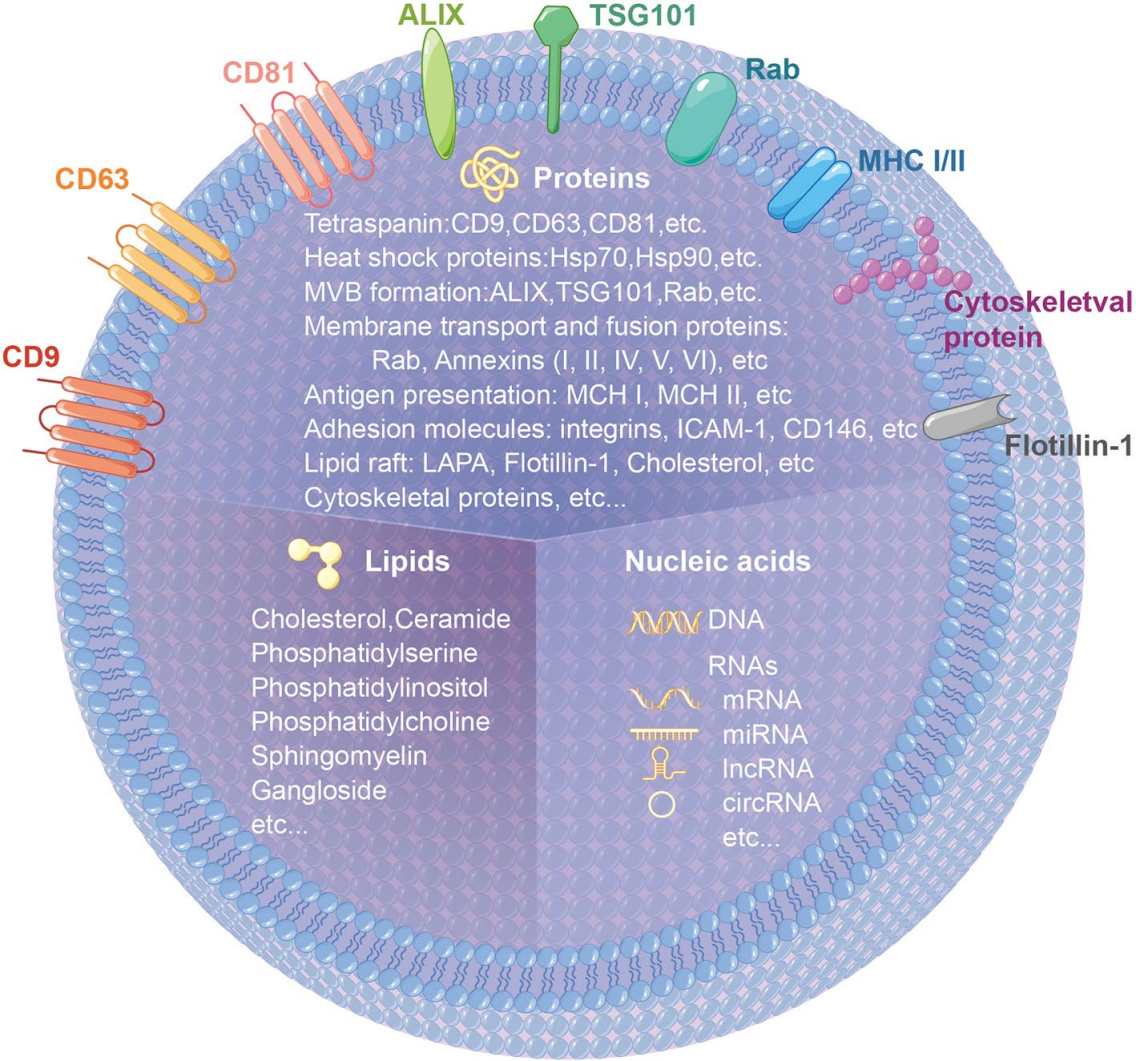
The hallmarks and cargos of exosomes. Exosomes are hemispherical structures with lipid bilayer membrane under electron microscope. Exosomes are composed of various components, mainly including proteins, lipids and nucleic acids

As observed under the electron microscope, exosomes are hemispherical structures with a lipid bilayer membrane [28]. Exosomes are composed of various components, mainly including proteins, lipids and nucleic acids (Table 1), and are abundantly present in body fluids, including blood, tears, urine, saliva, milk and ascites [38] (Fig. 1). Proteins mainly include tetraspanin, heat shock proteins, MVB formation, membrane transport and fusion proteins, antigen presentation, adhesion molecules, lipid raft and cytoskeletal proteins, which participate in the fusion of cell membranes and release of exosomes [29–31]. Lipids mainly include cholesterol, ceramide, phosphatidylserine, phosphatidylinositol, phosphatidylcholine, sphingomyelin and ganglioside, which are involved in the biological activity of exosomes [32–34]. Nucleic acids mainly include DNA, mRNA, miRNA, long non-coding RNA (lncRNA) and circular RNA (circRNA), which participate in the transmission of genetic information and diagnosis of diseases [35–37]. The specific components of exosomes are displayed in Table 1.

**Table 1:** Composition of exosomes

Type		Cargoes	Reference
Protein	Tetraspanin	CD9, CD63, CD81, etc	[29–31]
	Heat shock protein	Hsp70, Hsp90, etc	
	MVB formation	ALIX, TSG101, etc	
	Membrane transport and fusion proteins	Rab, Annexins (I, II, IV, V, VI), etc	
	Antigen presentation	MCH I, MCH II, etc	
	Adhesion molecules	integrins, ICAM-1, CD146, etc	
	Lipid raft	LAPA, Flotillin-1, Cholesterol, etc	
	Cytoskeletal proteins		
Lipids	Cholesterol, ceramide, phosphatidylserine, phosphatidylinositol, phosphatidylcholine, sphingomyelin, ganglioside, etc		[32–34]
Nucleic acids	DNA, mRNA, miRNA, lncRNA, circRNA, etc		[35–37]

### Formation of exosomes

**Fig. 2:**
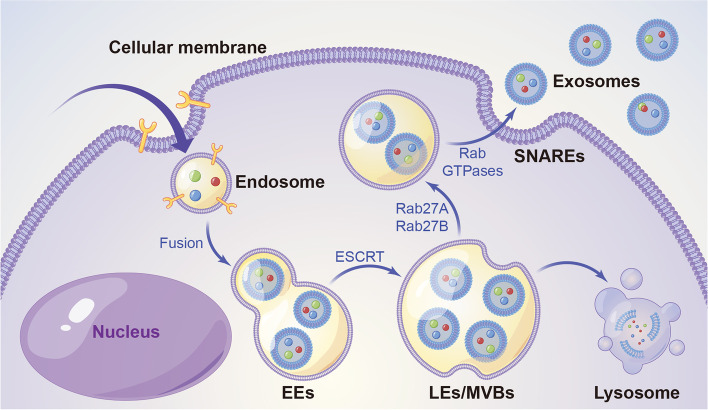
Exosome biogenesis and secretion within endosomal system by the endosomal sorting complex required for transport (ESCRT) pathway. Early endosomes (EEs) are formed by the fusion of endsomes. Subsequently, EEs depend on ESCRT to form multivesicular late endosomes (LEs)/bodies (MVBs). Rab27a and Rab27b direct the movement of LEs/MVBs toward the cell periphery, the SNARE complex helps LEs/MVBs fuse with the plasma membrane to release exosomes, and the rest of LEs/MVBs are degraded by lysosomes

The exact mechanism of exosome formation remains poorly understood, and the endosomal sorting complex required for transport (ESCRT) is a classical pathway [39, 40] (Fig. 2). The two main steps in the formation of exosomes are as follows: First, the cell membrane sags inward to form the early endosomes with accumulated luminal vesicles (ILV), and the endosomes are wrapped with proteins, lipids and nucleic acids synthesised by the cells; the endosomal membrane is depressed to bud inward to form tubular vesicles (intraluminal vesicles), that is, early endosomes (EEs) [41, 42]. Subsequently, the depressed membrane matures into multivesicular bodies (MVBs) with dynamic subcellular structures, that is, late endosomes (LEs), which can expose the transmembrane protein domain of the cytoplasm and release multiple vesicle structures into the extracellular environment upon fusion with the plasma membrane to form exosomes. Rab27a and Rab27b direct the movement of LEs/MVBs toward the cell periphery, the SNARE complex helps LEs/MVBs fuse with the plasma membrane to release exosomes, and the rest of LEs/MVBs are degraded by lysosomes [43, 44].

### Secretion of exosomes

Exosomes are secreted extracellularly through exocytosis upon the fusion of intercalated compartments with plasma membrane, which is the most basic and common process in cells [45]. However, in T cells and mast cells, this fusion is dependent on calcium ions for activation [46]. Most intracellular membrane fusions occur through specific protein mechanisms, such as N-ethylmaleimide-sensitive factor (NSF) for soluble factors and soluble NSF adhesion protein (SNAP) and SNAP adhesion protein receptor (SNARE) for membrane complex factors [47]. The two membranes in which fusion occurs should contain the corresponding SNAREs, namely vesicular SNARE (v-SNARE) and target SNARE (t-SNARE) [48, 49]. In addition, exosome secretion is controlled by Ras-associated GTP-binding protein 27a (Rab27a) and Rab27b [50, 51]. Synaptic binding protein-like 4 (SYTL4) and exophilin 5 (EXPH5) can inhibit Rab27a and Rab27b, leading to exosome secretion [51]. The exact mechanism of regulation of exosome secretion remains unclear, and the role of the above-mentioned molecules in exosome secretion requires further investigation.

### Function of exosomes

Exosomes are released by different cell types and can regulate the biological activity of target cells by transporting proteins, lipids and nucleic acids. They play a role in various biological processes such as angiogenesis, antigen presentation, apoptosis and inflammation [17]. They act by transferring informative substances, thus influencing physiological and pathological processes involved in cancer, neurodegenerative diseases, infections and autoimmune diseases [52–56]. Exosomes affect the recipient cells through two pathways [57]. The first pathway involves ligand–receptor interactions between exosomes and recipient cells, without internalising the exosome or its contents into the target cell. This pathway can regulate the activation or inhibition of target cell signalling pathways. The second pathway involves the entry of exosomes into cells through membrane fusion or endocytosis, wherein their components are taken up and released into the cytoplasm, thus affecting the host cells by regulating specific gene expression and signalling pathways and ultimately leading to changes in the cell function or phenotype.

### Detection of exosomes

In recent years, with the progress of research on exosomes in tumours, various technologies for exosome detection have been introduced that focus on the following three aspects: isolation and enrichment, identification and content analysis [58–62]. In addition, some researchers have developed various kits for the diagnosis and prognostic risk assessment of tumours based on the composition of exosomes [56, 63–66]. Development of such detection kits is a major clinical breakthrough in the field of early tumour diagnosis and provides an effective test for clinical diagnosis and the assessment of efficacy. However, there is a lack of a unified gold-standard method for exosome detection, which makes it difficult to be widely promoted in clinical settings. Therefore, it is necessary to discover a uniform and clinically recognised exosome detection technology.

## Exosomes and kidney cancer

### Involvement in the formation of tumour microenvironment

**Fig. 3:**
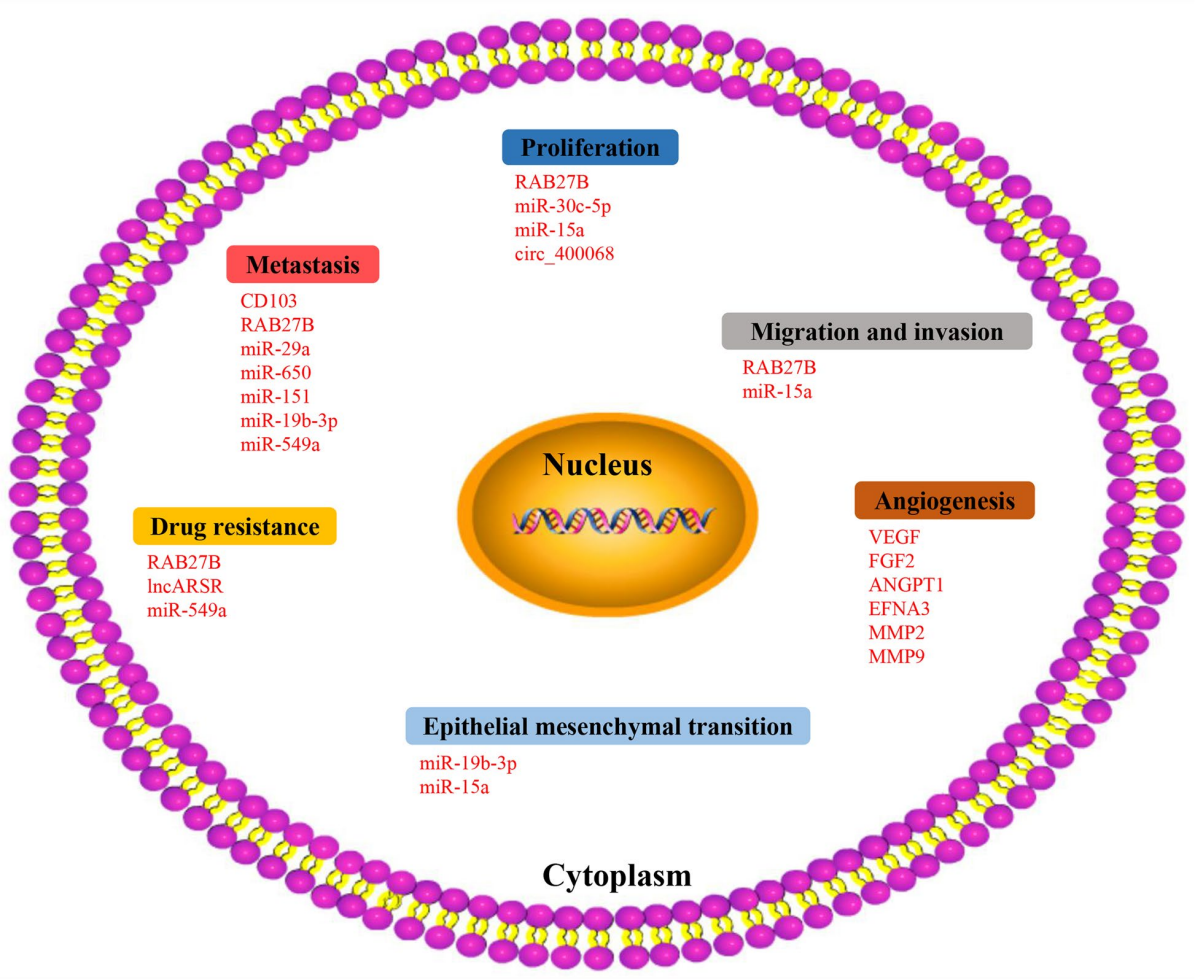
Role of exosomal constituents in kidney cancer. Exosomal component sare involved in the proliferation, migration and invasion, metastasis, angiogenesis, drug resistance, and epithelial mesenchymal transition (EMT) of kidney cancer

The tumour microenvironment is a key factor in the formation of tumours, and tumour cells can interact with their microenvironment to promote tumorigenesis and progression [67]. Exosomes exhibit certain characteristics of tissue and organ cellophilia, and the expression of this tendency is related to the expression of integrins on the surface of exosomes [68]. The establishment of pre-metastatic ecological niche is a complex process that involves the binding of exosomes secreted by cancer cells to the stromal cells of target organs, leading to the reprogramming of target cells, activation of signalling pathways and ultimately the establishment of a pre-metastatic microenvironment in target organs, thus providing the prerequisite for tumour metastasis [69, 70]. Exosomes are considered the main mediators of cell–cell interactions in the tumour microenvironment and are involved in promoting tumour cell invasion, angiogenesis and immunosuppression [71–75]. The role of exosomal constituents in kidney cancer are shown in Fig. 3.

### Contribution to angiogenesis

During tumorigenesis, tumour cells require a large supply of nutrients and oxygen to maintain rapid cell growth and reproduction. The formation of new blood vessels in the primary tumour foci provides more nutrients for the growth and spread of tumour cells [76, 77]. Tumour cells promote angiogenesis by activating endothelial cells [78]. Endothelial cells secrete exosomes rich in vascular endothelial growth factor (VEGF), fibroblast growth factor (FGF), angiopoietin-1 (ANGPT1), ephrin A3 (EFNA3), matrix metallopeptidase 2 (MMP2), matrix metallopeptidase 9 (MMP9) and azurocidin 1 (AZU1), which can stimulate the production of adjacent tumour blood vessels [78–82]. Grange et al. [70] verified that a subset of CD105-expressing tumour-initiating cells in human kidney cancer released microvesicles, which triggered angiogenesis and promoted the formation of pre-metastatic niches. Hou et al. [72] demonstrated that oncogenic miR-27a delivered by exosomes can bind to secreted frizzled-related protein 1 (SFRP1) and promote angiogenesis in kidney cancer. Tyrosine kinase inhibitor (TKI)-resistant kidney cancer can secrete low levels of exosomal miR-549a to induce vascular permeability and angiogenesis to promote kidney cancer metastasis [83]. Li et al. [84] found that ApoC1 transfer from kidney cancer cells to vascular endothelial cells through exosomes promoted angiogenesis and enhanced the migration and invasion of human umbilical vein endothelial cells (HUVEC) cells by activating signal transducer and activator of transcription 3 (STAT3). In addition, exosomes with high expression of carbonic anhydrase IX (CA IX) are associated with kidney cancer revascularisation [85]. The establishment of a vascular network is not only essential for the normal growth of tumour tissues but also provides an important channel for tumour invasion [86].

### Contribution to immune escape

Myeloid-derived suppressor cells (MDSCs) exert potent inhibitory effects on several immune cells, and their high concentration aggregation in the tumour microenvironment is one of the reasons for the formation of tumour immune escape [87, 88]. It was found that Hsp70 was abundantly present in exosomes secreted by mouse kidney cancer cells (Renca cells), upregulated the expression of arginase 1 (ARG-1), iNOS, interleukin 6 (IL-6) and VEGF and induced the expression of MDSCs by phosphorylating STAT3 (p-STAT3) pathway, thus promoting tumour growth [75, 89].

Natural killer (NK) cells are the main host defence factors against kidney cancer cells and can exert anti-tumour effects by either directly mediating cytotoxic activity through degranulation or promoting anti-tumour activity and producing immunomodulatory cytokines [90–92]. Xia et al. [93] found that exosomes of kidney cancer origin induced defective NK cell function through transforming growth factor-beta (TGF-β)/SMAD signalling pathway to evade natural immunity.

Exosomes secreted by kidney cancer cells can induce immune responses in T cells to trigger apoptosis of activated T lymphocytes by activating the caspase pathway. They can diminish the cytotoxicity of NK cells and reduce the production of IL-2, interferon gamma (IFN-γ), IL-6 and IL-10, which contribute to the immune escape and promote the development of kidney cancer [77, 94, 95]. In addition, exosomes isolated from human renal adenocarcinoma ACHN cells contain Fas ligands, which inhibit the action of the human immune system by inducing apoptosis of CD8+ T cells and ultimately help cancer cells in achieving immune escape [96].

### Involvement in cancer cell invasion and metastasis

A key molecular event in the development of target organ/tissue metastasis by tumours is the formation of tumour pre-metastatic niches [97]. Tumour pre-metastatic niches are defined as some molecular and cellular changes in metastatic-designated organs/tissues that can facilitate the colonisation of target organs/tissues by circulating tumour cells and promoting distant tumour metastasis [98]. In recent years, secretory components and cells found in distant metastatic tissues of different tumour animal models, including soluble factors, exosomes, vesicles and MDSCs, have confirmed the presence of tumour pre-metastatic niches in most types of malignancies [99, 100]. Exosomes can alter the target cell function through substances they carry; tumour-derived exosomes that act on epithelial cells lead to epithelial–mesenchymal transition (EMT), which is important in tumour metastasis [101].

## Role of exosomes in the diagnosis of kidney cancer

### Proteins

Raimondo et al. [73] identified 261 and 186 proteins by isolating urinary exosomes from normal patients and patients with kidney cancer, respectively, and most proteins were membrane-associated or cytoplasmic. Among these proteins, the expression of MMP9, ceruloplasmin (CP), podocalyxin like (PODXL), carbonic anhydrase IX (CAIX) and dickkopf 4 (DKK4) in urinary exosomes was higher in patients with kidney cancer than that in normal patients, and the expression of CD10, extracellular matrix metalloproteinase inducer (EMMPRIN), dipeptidase 1 (DPEP1), syntenin 1 and aquaporin 1 (AQP1) in urinary exosomes was higher in normal patients than that in patients with kidney cancer. These proteins may serve as potential markers of kidney cancer. Wang et al. [68] investigated the effect of exosomes isolated from cancer stem cells (CSCs) of 76 patients with metastatic RCC and 133 patients with localised RCC and found that CD103+ played a role in directing CSC exosomes to target cancer cells and organs. In addition, Tsuruda et al. [102] found that Rab27b protein can play an oncogenic role in renal cancer and sunitinib resistance through exosome-independent function.

### mRNA

mRNAs are a class of single-stranded RNAs that carry genetic information and can direct protein synthesis; they are transcribed from a strand of DNA as a template [103, 104]. Exosomes can carry and transport large amounts of mRNA to function in the recipient cells [23]. Grange et al. [70] identified mRNAs implicated in tumour progression and metastasis through molecular characterisation of microvesicles, including VEGF, FGF2, ANGPT1, EFNA3, MMP2 and MMP9. In addition, Palma et al. [105] reported that the mRNA levels of glutathione s-transferase alpha 1 (GSTA1), CCAAT enhancer binding protein alpha (CEBPA) and pterin-4 alpha-carbinolamine dehydratase 1 (PCBD1) in urinary extracellular vesicles were lower in patients with RCC than those in controls, and the mRNA levels of these three genes returned to normal 1 month after nephrectomy. This demonstrates that mRNA levels in urinary extracellular vesicles serve as potential molecular markers for the diagnosis of RCC.

### miRNA

miRNAs are smaller endogenous non-coding RNAs (18–24 nucleotides) that regulate protein translation after gene transcription [106, 107]. They can act as oncogenes or tumour suppressors involved in tumorigenesis [108, 109]. Several exosomal miRNAs have been identified to be differentially expressed in patients with renal cancer and normal patients. Grange et al. [70] found that 24 miRNAs, including miR-200c and miR-650, were significantly upregulated in CD105+ microvesicles, and 33 miRNAs, including miR-100 and miR-296, were significantly downregulated, and several miRNAs such as miR-29a, miR-650, and miR-151 were associated with tumour invasion and metastasis. Zhang et al. [110] found that the expression levels of miR-210 and miR-1233 in blood exosomes were significantly higher in patients with RCC than those in healthy subjects, and the expression levels were significantly decreased after surgical removal of the tumour. Xiao et al. [111] sequenced exosomal miRNAs from plasma samples and found that the expression level of miR-149-3p and miR-424-3p was upregulated, whereas that of miR-92a-1-5p was significantly decreased. In addition, other miRNAs were reported to be potential diagnostic biomarkers of kidney cancer [68, 83, 112–118].

### lncRNA

lncRNAs are RNAs that are longer than 200 nucleotides and cannot code for proteins [119]. They can control cellular transcription and protein translation by interacting with proteins, mRNAs or miRNAs [120]. Malignant tumour cells can express specific lncRNA markers, indicating that lncRNAs can be used as disease-specific markers that are important for cancer diagnosis [121]. lncRNAs are abundantly expressed in exosomes and can be protected by the exosomal tegument with higher stability [122, 123]. Similar to miRNAs, lncRNAs play an important role in the growth, proliferation, invasion and metastasis of cancer cells [124]. Qu et al. [125] demonstrated that exosome-transmitted lncARSR promoted AXL and c-MET expression in RCC cells by competitively binding to miR-34/miR-449, thereby promoting sunitinib resistance. Exosomal lncRNAs are important in tumour biology, and further studies are required to understand the role of exosomal lncRNAs in renal cancer.

### circRNA

CircRNAs are a newly discovered type of non-coding RNAs that form a covalently closed continuous loop structure that originates from exons or introns by specific selective shearing [126–128]. It has been found that a large number of circRNAs can be detected in exosomes. circRNAs function as miRNA sponges during gene regulation [129, 130]. Based on the circRNA expression array data, Xiao et al. [131] found that circ_400068 was significantly upregulated in exosomes derived from RCC. At present, circRNAs in exosomes derived from renal cancer cells have been investigated in a relatively small number of studies, and therefore, further investigation is required. Potential biomarkers derived from exosomes that have been validated in kidney cancer are listed in Table 2. Figure 3 demonstrates the role of exosomal constituents in kidney cancer.

**Table 2:** Exosomes derived potential biomarker for kidney cancer

Type	Source	Cohorts	Method	Cargoes	Year	Reference
Protein	Urine	29 RCC, 23 health	LC-MS/MS, western blotting	MMP9, CP, PODXL, CAIX and DKK4; CD10, EMMPRIN, DPEP1, Syntenin 1 and AQP1	2013	Raimondo et al. [73]
	Serum	76 metastatic RCC, 133 located RCC	Flow cytometry	CD103	2019	Wang et al. [68]
	Supernatants	A498 cell	ExoELISA-ULTRA CD63 kit	RAB27B	2020	Tsuruda et al. [102]
mRNA	Supernatants	Cancer stem cells	Microarray, qRT-PCR	VEGF, FGF2, ANGPT1, EFNA3, MMP2 and MMP9	2011	Grange et al. [70]
	Urine	46 RCC, 22 health	Microarray, qRT-PCR	GSTA1, CEBPA, PCBD1	2016	Palma et al. [105]
miRNA	Supernatants	Cancer stem cells	Microarray, qRT-PCR	miR-200c, miR-650, miR-100, miR-296, miR-29a, miR-650, miR-151	2011	Grange et al. [70]
	Urine	81 RCC, 33 health	Microarray, qRT-PCR	miR0126-3p, miR-449a, miR-34b-5p, miR-486-5p, miR-30c-5p	2016	Butz et al. [118]
	Plasma	44 metastatic RCC, 65 metastatic RCC	RNA-sequencing, qRT-PCR	miR-let-7i-5p, miR-26a-1-3p, miR-615-3p	2017	Du et al. [113]
	Serum	82 RCC, 80 health	qRT-PCR	miR-210, miR-1233	2018	Zhang et al. [110]
	Supernatants	HK-2 cell, 786-O cell	qRT-PCR	miR-205	2018	Crentsil et al. [115]
	Serum	40 RCC, 30 health, 5 lung metastasis RCC	Microarray, qRT-PCR	miR-210	2018	Wang et al. [117]
	Serum	76 metastatic RCC, 133 located RCC	qRT-PCR	miR-19b-3p	2019	Wang et al. [68]
	Urine	70 early-stage RCC, 30 health	NGS, qRT-PCR	miR-30c-5p	2019	Song et al. [112]
	Urine/ Supernatants	Mice urine; mouse and human tRCC cell lines	qRT-PCR	miR-204-5p	2019	Kurahashi et al. [114]
	Plasma	5 RCC, 5 health; 22 RCC, 16 health	RNA-sequencing; qRT-PCR	miR-92a-1-5p, miR-149-3p, miR-424-3p	2020	Xiao et al. [111]
	Supernatants	786-O cell, 786-O-SR cell	qRT-PCR	miR-549a	2021	Xuan et al. [83]
	Plasma	ACHN cell	Microarray, qRT-PCR	miR-15a	2021	Li et al. [116]
lncRNA	Plasma	71 advanced RCC, 32 health	qRT-PCR	lncARSR	2016	Qu et al. [125]
circRNA	Plasma	28 RCC	circRNA microarray	circ_400068	2020	Xiao et al. [131]

## The role of exosomes in kidney cancer treatment

### Tumour drug resistance

Tumour drug resistance is one of the main reasons for the failure of clinical treatment of tumours. Drug-resistant tumour cells can secrete exosomes that contain the genetic information of multiple drug resistance-associated proteins, which in turn cause other tumour cells to acquire drug resistance [132, 133]. Several receptor tyrosine kinases associated with angiogenesis and tumour microenvironment are overexpressed mainly owing to the inactivation of Von Hippel–Lindau (VHL) tumour suppressor genes in renal cancer; therefore, TKIs, including sunitinib, have become one of the first-line therapies for renal cancer [134]. However, sunitinib resistance has made the clinical benefit of sunitinib treatment limited at present [135]. Qu et al. [125] found that drug-resistant cells in nephropathy transmitted lncARSR to other cells through exosomes, causing them to develop drug resistance, and lncARSR promoted AXL/c-MET expression by competitively binding to miR-34/miR-449. MET expression, which in turn promoted lncARSR expression as positive feedback, further promoted drug resistance in renal cancer cells. In addition, Tsuruda et al. [102] found that Rab27b can play an oncogenic role in sunitinib resistance in renal cancer through exosome-independent function. The above-mentioned study demonstrates that exosomes mediate the development of drug resistance in tumour cells, which can not only provide novel therapeutic targets for patients but also predict the responsiveness of patients to anti-tumour drugs through the detection of exosomal markers, thus providing an important reference for individualised treatment of kidney cancer [44, 136].

### Drug carriers

Owing to their lipid bilayer membrane structure, exosomes can protect RNA present inside the membranes from degradation by RNA enzymes, and owing to their smaller particle size and deformability, they can cross the biological membranes more easily, thus facilitating precise delivery of therapeutic genes to the target cells [137, 138]. Exosomes can mediate the transfer of genetic material, thus altering the biological activity of recipient cells [139]. Exosomes can carry various therapeutic substances, including RNAs and antisense oligonucleotides [24]. Exosomes can deliver therapeutic substances directly to target organs through different biological barriers, for example, macrophage-derived exosomes can effectively cross the blood–brain barrier to deliver protein-like substances [140]. Ligand enrichment on engineered exosomes can also be used to induce or inhibit signalling in the receptor cells for targeting exosomes to specific cells [141]. In addition, exosomes can be effectively loaded with chemotherapeutic drugs with low toxic side effects. Therefore, they can serve as well-tolerated and promising drug carriers [142, 143]. Currently, exosomes are considered important drug delivery carriers for the treatment of cardiovascular diseases and pancreatic cancer [35, 144]; however, their role in kidney cancer requires further investigation [145].

### Tumour vaccines

Compared with conventional vaccines, the vaccines developed using exosomes derived from tumour cell secretion may exert incomparable effects with higher affinity [146]. Exosomes secreted by tumour cells can present tumour-associated antigens and induce the development of immunity against tumours [94]. Zhang et al. found that IL-12-anchored kidney cancer cell-derived exosomes induced the production of more cytotoxic T lymphocytes specific for kidney cancer antigens and improved anti-tumour effects [147]. They further constructed an enhanced immunogenic EXO-IL-12 vaccine capable of stably expressing kidney cancer-specific antigen G250, immune-associated protein and GPI-IL-12, which can significantly enhance the proliferation and activation of T lymphocytes in vitro and exert an induced antigen-specific killing effect [74, 148]. Another study found that mice with kidney cancer vaccinated with tumour exosome-loaded dendritic cell (DC-TEX) vaccine had a longer survival period than that of mice vaccinated with tumour cell lysate-loaded dendritic cell vaccine [149]. Exosomes that are loaded and delivered with tumour suppressor genes that inhibit tumour cell growth provide necessary conditions for the development of exosomal tumour vaccines [150–152].

## Conclusion

Early diagnosis of kidney cancer is one of the key factors in improving the survival rate of patients. Exosomes may benefit early diagnosis. Exosomes secreted by kidney cancer cells are abundantly present in blood, urine and other body fluids, thus providing advantages such as easy availability, non-invasive examination and tumour specificity. Owing to their small size, high mobility and lipid bilayer structure, they can easily passthrough biological membranes and protect rich bioactive substances present inside the membranes from degradation; therefore, exosomes have become a prime focus of research. Tumour-derived exosomes carry a large number of substances, including proteins, nucleic acids and lipids, which can alter the biological behaviour of target cells and participate in the development of kidney cancer. Numerous studies have found that the expression of exosomes is significantly different in patients with kidney cancer and normal subjects. Exosomes play an important role in the infiltration and metastasis of kidney cancer and also participate in tumour drug resistance and immune escape. Studies related to exosomes provide new ideas for the diagnosis and treatment of kidney cancer and offer adequate developmental prospects. However, studies on exosomes derived from renal cancer cells are mostly retrospective, and the tissue types mostly include renal clear cell carcinoma. To promote the application of exosomes in clinical settings, more extensive studies combined with clinical trials are required, and future studies should include increased sample sizes and different tissue types and adopt a prospective study design, which will be more convincing and provide substantial medical data support for clinical translation. In addition, the study of exosomes in kidney cancer is relatively independent and none of the molecules identified seem to have been repeatedly validated in different studies, which requires more prospective clinical trials leading to more reproducible biomarkers. Moreover, further investigation is required for developing exosome-mediated tumour vaccines and understanding the effect and mechanism of drug resistance on targeted therapy for kidney cancer.

## Data Availability

The datasets used and analysed during the current study are available from the corresponding author on reasonable request.

## Abbreviations

ALIX: ALG-2 interacting protein X
ANGPT1: Angiopoietin-1
AQP1: Aquaporin 1
ARG-1: Arginase 1
AZU1: Azurocidin 1
CA IX: Carbonic anhydrase IX
CEBPA: CCAAT enhancer binding protein alpha
circRNA: Circular RNA
CP: Ceruloplasmin
CSCs: Cancer stem cells
DKK: 4dickkopf 4
DPEP1: Dipeptidase 1
EEs: Early endosomes
EFNA3: Ephrin A3
EMMPRIN: Extracellular matrix metalloproteinase inducer
EMT: Epithelial–mesenchymal transition
ESCRT: Endosomal sorting complex required for transport
EXPH5: Exophilin 5
FGF: Fibroblast growth factor
FLOT1: Flotillin-1
GSTA1: Syntenin 1 and glutathione s-transferase alpha 1
Hsp: Heat shock proteins
HUVEC: Human umbilical vein endothelial cells
ICAM-1: Intercellular adhesion molecule-1
IFN-γ: Interferon gamma
IL-6: Interleukin 6
LEs: Late endosomes
lncRNA: Long non-coding RNA
MDSCs: Myeloid-derived suppressor cells
MHC: Major histocompatibility complex
miRNAs: microRNAs
MMP2: Matrix metallopeptidase 2
MMP9: Matrix metallopeptidase 9
mRNAs: Messenger RNAs
MVBs: Multivesicular bodies
NK: Natural killer
NSF: N-ethylmaleimide-sensitive factor
PCBD1: Pterin-4 alpha-carbinolamine dehydratase 1
PODXL: Podocalyxin like
Rab: Ras-associated GTP-binding
RCC: Renal cell carcinoma
SFRP1: Secreted frizzled-related protein 1
SNAP: Soluble NSF adhesion protein
SNARE: SNAP adhesion protein receptor
STAT3: Signal transducer and activator of transcription 3
SYTL4: Synaptic binding protein-like 4
TGF-β: Transforming growth factor-beta
TKI: Tyrosine kinase inhibitor
TSG101: Tumour susceptibility gene 101
t-SNARE: Target SNARE
VEGF: Vascular endothelial growth factor
VHL: Von Hippel–Lindau
v-SNARE: Vesicular SNARE
